# Purinergic Signaling in Liver Pathophysiology

**DOI:** 10.3389/fendo.2021.718429

**Published:** 2021-08-11

**Authors:** Shanu Jain, Kenneth A. Jacobson

**Affiliations:** Molecular Recognition Section, Laboratory of Bioorganic Chemistry, National Institute of Diabetes and Digestive and Kidney Diseases, Bethesda, MD, United States

**Keywords:** purinergic signaling, adenosine receptors, P2 receptors, hepatocyte, stellate cell

## Abstract

Extracellular nucleosides and nucleotides activate a group of G protein-coupled receptors (GPCRs) known as purinergic receptors, comprising adenosine and P2Y receptors. Furthermore, purinergic P2X ion channels are activated by ATP. These receptors are expressed in liver resident cells and play a critical role in maintaining liver function. In the normal physiology, these receptors regulate hepatic metabolic processes such as insulin responsiveness, glycogen and lipid metabolism, and bile secretion. In disease states, ATP and other nucleotides serve as danger signals and modulate purinergic responses in the cells. Recent studies have demonstrated that purinergic receptors play a significant role in the development of metabolic syndrome associated non-alcoholic fatty liver disease (NAFLD), non-alcoholic steatohepatitis (NASH), fibrosis, hepatocellular carcinoma (HCC) and liver inflammation. In this concise review, we dissect the role of purinergic signaling in different liver resident cells involved in maintaining healthy liver function and in the development of the above-mentioned liver pathologies. Moreover, we discuss potential therapeutic strategies for liver diseases by targeting adenosine, P2Y and P2X receptors.

## Introduction

The action of adenosine triphosphate (ATP) as an extracellular signaling molecule was initially proposed by Burnstock in 1972 (1, 2). ATP and its hydrolytic products (ADP and adenosine) along with other nucleotides and nucleotide sugars (UTP, UDP, UDP-glucose) act as extracellular signals to regulate various physiological and pathophysiological processes (3–7). These signaling molecules activate two families of purinoceptors: Adenosine receptors (also designated as P1) are activated principally by adenosine and consist of four subtypes shown as rodent gene name (subtype name): Adora1 (A_1_AR), Adora2a (A_2A_AR), Adora2b (A_2B_AR), Adora3 (A_3_AR). The adenosine receptors differ in their affinity for adenosine, with A_1_AR and A_2A_AR exhibiting high affinity and A_3_AR and A_2B_AR having lower affinity (8). A_1_AR and A_3_AR couple to G_i_/_o_ proteins, whereas A_2A_AR and A_2B_AR couple to G_s/olf_ proteins causing a decrease or increase in intracellular cAMP levels upon receptor activation respectively. P2 receptors are divided into ionotropic ligand-gated ion channel P2X (P2X1-7, gene name P2rx#), principally activated by ATP, and metabotropic G protein-coupled P2Y (P2Y_1,2,4,6,11,12,13,14_, gene name P2ry#) receptors. P2Y_1,2,4,6,11_ receptors belong to the P2Y_1_-like subfamily and couple to G_q/11_, G_o_, G_12/13_, G_s_ protein, whereas P2Y_12,13,14_ receptors are classified as P2Y_12_-like and couple to G_i/o_ protein, thereby activating different intracellular signaling pathways.

The liver is the largest internal organ in the body with a diverse range of functions including metabolism of glucose and other carbohydrates along with lipids, protein synthesis, detoxification, and bile secretion (9). The liver is a major site for nucleotide synthesis (salvage or *de novo* synthesis), and the release of these nucleotides into extracellular space may result in autocrine and paracrine activation of purinergic receptors on different cells, regulating various biological processes (10–12). ATP release can be mediated by various mechanisms such as exocytosis from lysosomes, unregulated release from necrotic cells, co-release with hormones, controlled release through connexin and pannexin hemichannels, and P2X7 ion channels (13–18). In this concise review, we will discuss the understanding of purinergic signaling in liver physiological and pathophysiological processes. We will also briefly describe potential clinical applications of purinergic signaling-based drugs for the therapeutics of liver disorders.

## Sources of Extracellular Nucleotides and Adenosine in Liver

Adenosine is produced in the extracellular space *via* dephosphorylation of ATP by a two-step enzymatic reaction sequence. First, CD39 (ecto-nucleoside triphosphate diphosphohydrolase 1: ENTPD1, NTPDase1) and NTPases convert ATP or ADP to AMP. In the second step, hydrolysis of AMP by CD73 (ecto-5’-nucleotidase: NT5E) results in the generation of the adenosine. The ectonucleotide pyrophosphatase/phosphodiesterase (ENPP) family is also responsible for hydrolysis of extracellular nucleotides. The CD39-CD73-adenosine axis has been implicated in liver immune responses and inflammation related to various diseases (19). Intracellularly generated adenosine can also be transported across cell membranes by ENTs (equilibrative nucleoside transporters) and CNTs (concentrative nucleoside transporters). Vesicular nucleotide transporter (VNUT, SLC17A9) expressed by mouse hepatocytes has been shown to promote vesicular release of ATP and other nucleotides (20). VNUT-dependent ATP release from the hepatocytes triggered postprandial triglyceride release and aggravated steatohepatitis in the liver (20). The authors also demonstrated that high blood glucose stimulated the release of ATP from hepatocytes, and this phenomenon was inhibited in mice lacking VNUT (20). Treatment of a mouse model of NASH with VNUT inhibitor (clodronate) reduced hepatic inflammation, fibrosis, and triglyceride accumulation (21). Another study demonstrated that human hepatocytes can release ATP in response to cell swelling or osmotic stress (22). Autocrine purinergic signaling mediated by ATP led to Cl^-^ secretion that helped to recover the cell volume (22). Intrahepatic mechanical stress induced during hepatectomy resulted in the robust release of ATP from a lysosomal compartment of hepatocytes and Kupffer cells (23). This increased extracellular ATP levels promoted liver regeneration in the rat post-surgery (23). Connexin hemichannels required for the release of ATP have been implicated in steatohepatitis (24). Treatment of mice with connexin inhibitors (TAT-Gap24 and TAT-Gap19) decreased inflammatory markers and liver lipid levels while increasing superoxide dismutase levels (24). Another study demonstrated the role of pannexin1 (PANX1) in the pathogenesis of liver diseases (25). The mice lacking PANX1 displayed reduced inflammation compared to the control mice when induced with steatohepatitis (25). Selective deletion of CD39 has been studied by Robson and others (26). CD39 is beneficial during liver regeneration and for hepatic glucose metabolism. However, CD39 deletion in natural killer (NK) cells reduces interferon-g production to attenuate ischemia/reperfusion injury in mouse liver. Hence, extracellular nucleotides and purinergic signaling have been implicated in the regulation of various hepatic processes (26–29).

## Liver - a Metabolic Organ

The liver is a key organ for the regulation of glucose homeostasis in both fed and fasted conditions (30, 31). During fasting, stored glycogen in the liver is broken down by the process of glycogenolysis to maintain normoglycemia. The liver also contributes to fasting glucose production by the process of gluconeogenesis. The net hepatic glucose output helps to provide an energy source to extrahepatic tissues during starvation (30, 31). Starvation also promotes the accumulation of lipids (triacylglycerol (TAG) and diacylglycerol (DAG)) in the liver. These key liver functions are mainly performed by parenchymal cells termed hepatocytes (30). Hepatocytes make up to roughly 80% of the total hepatic mass. In addition to the parenchymal cells, the liver contains non-parenchymal cells, i.e. hepatic stellate cells (HSC, fat-storing pericytes located between a sinusoidal capillary and hepatocytes), cholangiocytes (bile duct epithelial cells) and Kupffer (resident macrophages), vascular endothelial and smooth muscle cells, that through crosstalk with hepatocytes and with each other regulate liver functions (31, 32). Many non-resident cells infiltrating into the liver such as macrophages, neutrophils, dendritic cells, natural killer cells, and T and B lymphocytes regulate cytokine production affecting liver metabolic activity in pathophysiological conditions (32). Most of the liver cell types (both resident and infiltrating) express multiple purinergic receptor subtypes (32).

## Hepatic Carbohydrate and Lipid Metabolism

Purinergic signaling plays a role in various processes related to carbohydrate and lipid metabolism in the liver. Mechanical stimulation, stress such as hypoxia, or cell lysis may cause the release of nucleotides, such as ATP and UTP, by hepatocytes (and consequently elevated adenosine) that induce Ca^2+^-mediated glycogenolysis in neighboring hepatocytes (11). Extracellular ATP stimulates glycogenolysis in hepatocytes and perfused livers (33–36). Treatment of human hepatocytes with BzATP, a P2XR agonist, decreases glycogen content (37). Mechanistically, stimulation of P2X resulted in Ca^2+^-mediated activation of glycogen phosphorylase, a rate-limiting enzyme in the glycogenolysis pathway (37–39). In the perfused liver, UTP induces glycogenolysis more potently than ATP due to its robust effect on thromboxane secretion from the non-parenchymal cell (40). ATP also enhances Ca^2+^-mediated gluconeogenesis in hepatocytes (41, 42). However, high concentrations of ATP inhibit gluconeogenesis from sources such as pyruvate and lactate (43). Stimulation of cultured hepatocytes by ATP attenuates glycolysis, through inhibition of phosphofructokinase-2 (44). Activation of A_1_AR enhanced Ca^2+^-mediated glycogenolysis in isolated rat hepatocytes (45). The authors also showed that activation of A_2A_R with a selective agonist (CGS21680) also promoted glucose release *via* gluconeogenesis in rat hepatocytes (45).

Nucleotides also regulate liver lipid metabolism. Extracellular ATP inhibits acetyl-CoA carboxylase (ACC) by elevating intracellular calcium levels in rat hepatocytes (46). ATP treatment also simultaneously inhibited carnitine O-palmitoyltransferase I (CPT-1) activity through a PKC-dependent mechanism (46). A_2A_R deficiency enhanced expression and activity of lipogenic gene-sterol regulatory element-binding protein 1c (SREBP1c) in mouse hepatocytes (47). Extracellular nucleotides were also reported to play a key role in reverse cholesterol transport. Chronic activation of P2Y_13_R by a partial agonist (AR-C69931MX) increased liver uptake of cholesterol (48). Further, a study on P2Y_13_R deficient mice displayed impaired features of reverse cholesterol transport, independent of plasma HDL levels (49, 50).

## Purinergic Signaling in Liver Metabolic Disorders

### Non-Alcoholic Fatty Liver Disease and Non-Alcoholic Steatohepatitis

Metabolic syndrome and non-alcoholic fatty liver disease (NAFLD) have a bidirectional mutual relationship, suggesting that the occurrence of one can enhance the severity of the other. The effect of metabolic syndrome on NAFLD may be greater than the effects of NAFLD on metabolic syndrome (51, 52). Metabolic syndrome characterized by obesity, insulin resistance, dyslipidemia, and glucose tolerance can initiate ectopic deposition of lipids in the liver causing NAFLD (52). NAFLD can progress to non-alcoholic steatohepatitis (NASH), a severe form of NAFLD associated with liver inflammation (53). Studies have been conducted showing the effects of purinergic receptors directly on liver dysfunction or indirectly *via* improving features of metabolic syndrome. The liver of streptozotocin (STZ)-induced diabetic rats showed increased adenosine A_1_AR expression (54). However, a different study claimed no change in A_1_AR expression, whereas expression of A_2A_AR and A_3_AR receptors was significantly upregulated in STZ-treated rat liver (55). P2X7R expression was increased in hepatocytes, Kupffer cells, and liver sinusoidal endothelial cells in the NASH disease model (56). Lack of P2X7R ameliorates hepatocyte apoptosis and decreases inflammation and fibrosis in mice treated with carbon tetrachloride (CCl_4_) with a high fat diet (HFD) (56, 57). Activation of P2X7R on Kupffer cells enhances the production of TNF-α and monocyte chemotactic protein-2 (MCP-2) production in HFD mice treated with CCl_4_ (57). These studies suggest that P2X7R antagonists may prove useful for the treatment of NASH.

**Figure 1:**
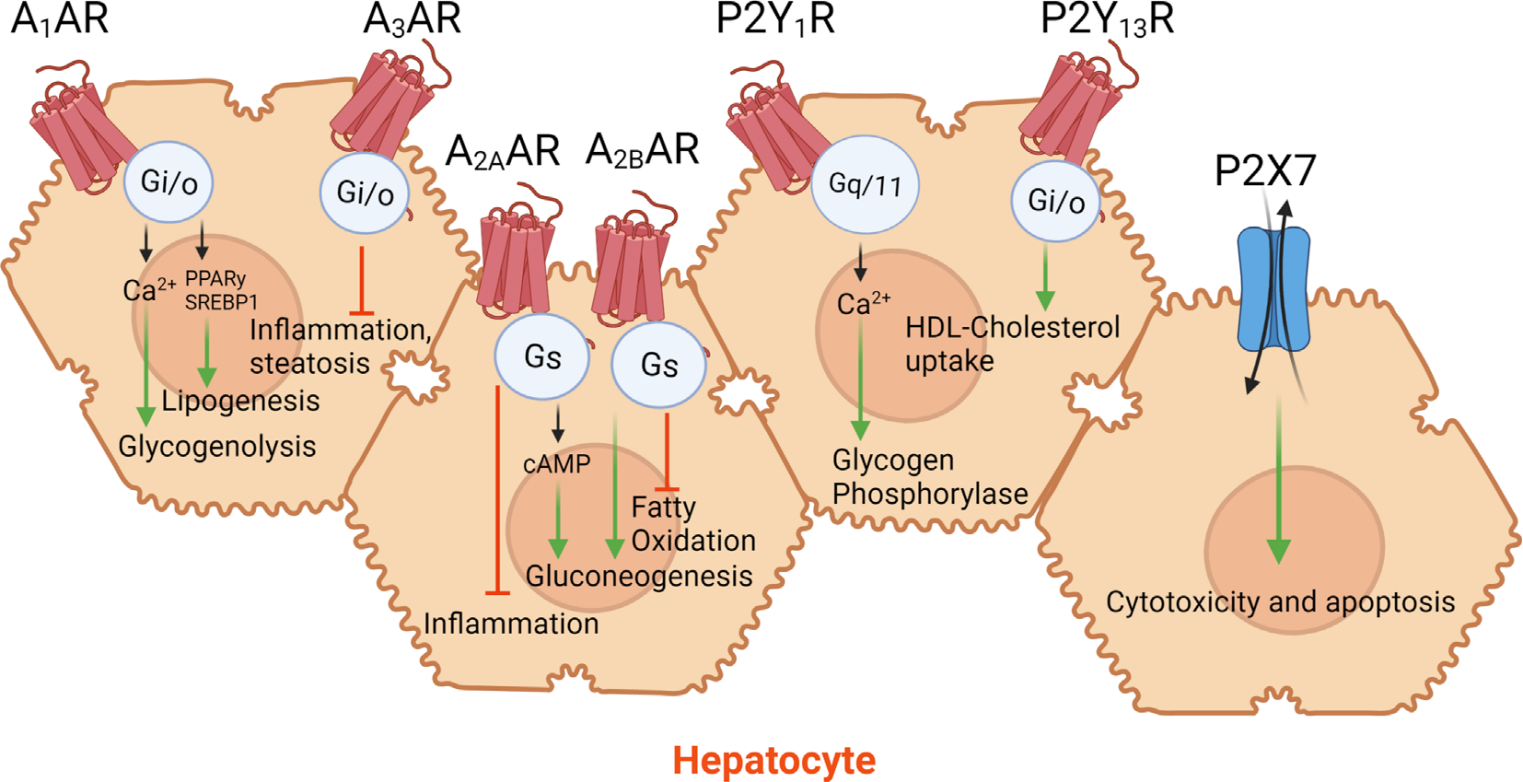
Purinergic signaling in hepatocytes. Hepatocytes are parenchymal cells of the liver and are involved in maintaining whole-body glucose and lipid homeostasis. Hepatocytes express various purinergic receptors that play a key role in regulating glucose, lipid, cholesterol metabolism, and hepatocyte apoptosis.

A_2A_AR activation has an anti-inflammatory effect (58, 59), whereas its deficiency increases pro-inflammatory responses (60). Further, the lack of whole-body A_2A_AR in mice enhanced HFD-induced NAFLD and liver inflammation (47). Accordingly, deficiency of A_2A_AR in hepatocytes and macrophages contributed to enhanced inflammation (47). The effect of A_2A_AR on inflammation was also demonstrated in the methionine- and choline-deficient (MCD)-induced NASH mouse model. The MCD-NASH mouse model combined with A_2A_AR knockout (KO) exhibited higher body weight, enhanced liver inflammation, and severe hepatic steatosis than the control group (61). The A_2A_AR’s role in reducing inflammation caused by lipotoxicity substantially imparted protection against the development of NASH (62, 63). These studies suggest the therapeutic potential of A_2A_AR agonists in decreasing inflammation associated with NAFLD/NASH and metabolic syndrome (**Figure 1**).

A_2B_AR was also demonstrated to play a critical role in regulating fatty liver disease. Deficiency of A_2B_AR protected mice from hepatic steatosis and development of fatty liver (64). Inhibition of A_2B_AR by selective antagonist ATL-801 in diabetic KKA(Y) mice reduced glucose output during hyperinsulinemic-euglycemic clamp studies (65). Some of the contrasting studies showed that A_2B_AR activation inhibited lipogenic genes such as sterol regulatory element-binding protein-1 (SREBP-1). HFD mice lacking A_2B_AR displayed hepatic steatosis with enhanced plasma triglyceride and cholesterol levels (66). Furthermore, overexpression and activation of hepatic A_2B_AR reduced lipid synthesis in the liver and improved whole-body metabolism (66). A_2B_AR KO mice on regular diet showed reduced weight and increased *de novo* lipogenesis resulting in elevated liver triglyceride levels. Increased mRNA levels of glucokinase and fatty acid synthase confirmed impaired lipid metabolism in the liver of A_2B_AR KO mice (67). HFD A_2B_AR KO mice exhibited impaired glucose tolerance and insulin sensitivity (68). Wild type (WT) mice treated with A_2B_AR agonist/partial agonist (BAY60-6553) displayed improved glucose and insulin tolerance and decreased fasting blood glucose levels (68). These observations render A_2B_AR a good drug target for the treatment of liver diseases (**Figure 1**).

Recent studies have highlighted the importance of the A_3_AR in NAFLD/NASH. A_3_AR expression in livers from NAFLD patients was decreased by 1.9-fold compared to controls, highlighting a plausible role of the receptor in NAFLD pathophysiology (69). Global deficiency of A_3_AR in mice fed a HFD enhanced expression of genes involved in hepatic inflammation and steatosis (69) (**Figure 1**). The authors showed that administration of an A_3_AR agonist prodrug (MRS7476, 5 mg/kg, p.o., b.i.d.) protected the STAM mouse model against the development of NASH (69). The two succinyl ester groups of MRS7476 greatly increase its water solubility and are likely cleaved in the gut, rather than the site of action. Another study showed the efficacy of A_3_AR agonist Cl-IB-MECA (namodenoson) in the treatment of NASH in mice (70). The drug namodenoson is currently in Phase 2 clinical trials for NASH therapeutics (ClinicalTrials.gov Identifiers: NCT02927314 and NCT04697810, accessed 05-31-2021).

**Figure 2:**
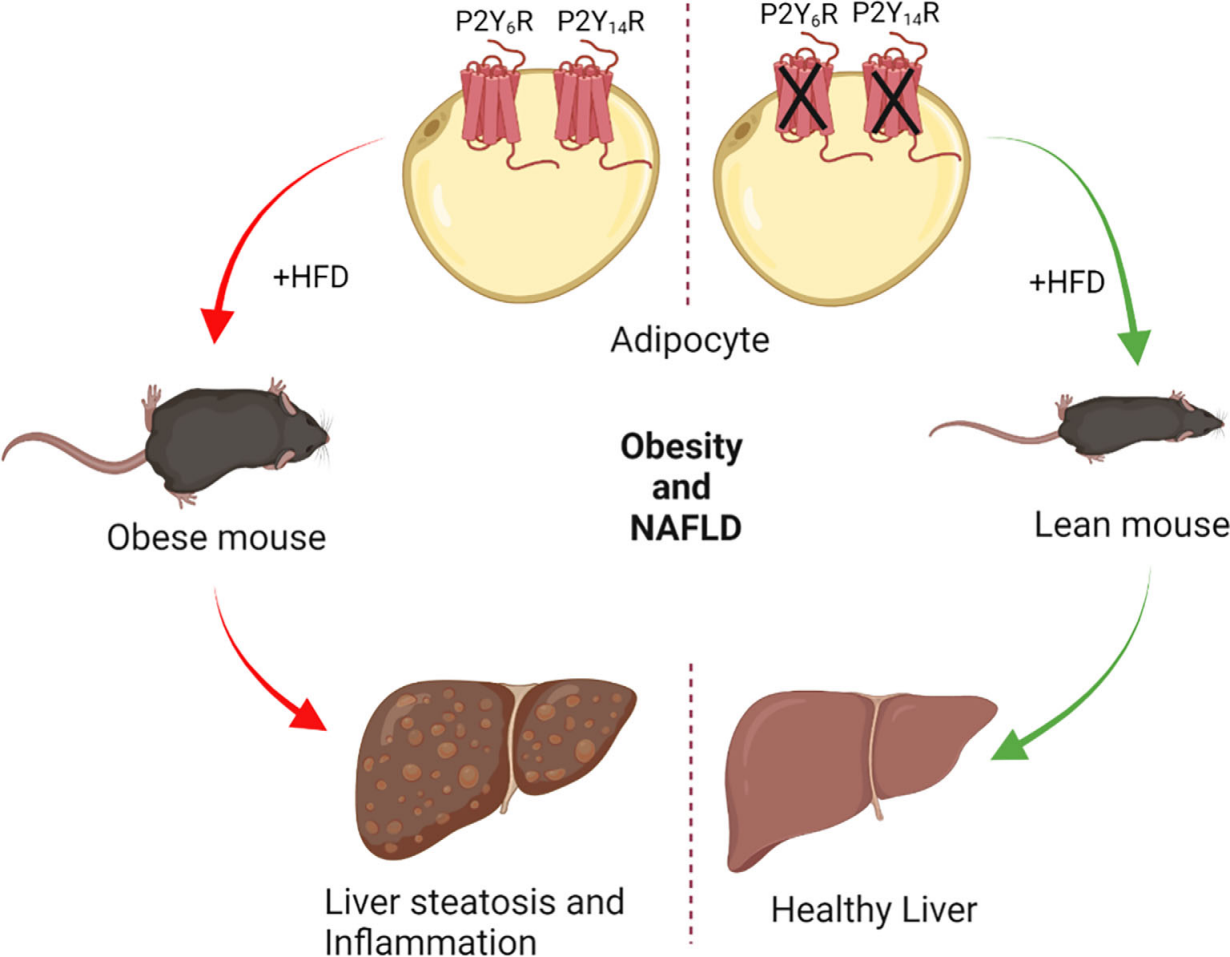
Dysregulated purinergic signaling in adipocytes alleviates obesity and associated NAFLD. Lack of P2Y_6_R or P2Y_14_R specifically in adipocytes protects against diet-induced obesity. Reduced fat mass prevented the ectopic deposition of lipids in the liver, decreasing hepatic steatosis. Hepatic inflammation was reduced in mice lacking P2Y_6_R or P2Y_14_R in adipocytes. Antagonists of P2Y_6_R and P2Y_14_R may prove beneficial for the treatment of obesity-associated NAFLD.

Obesity is a key risk factor for the development of NAFLD, and hence the mainstay treatment for NAFLD and NASH is weight loss. Recent studies have demonstrated the role of P2Y receptors in regulating obesity and its impact on liver steatosis and inflammation. Mice lacking P2Y_6_R selectively in adipocytes were protected from diet-induced obesity (without a significant change in food intake) and systemic inflammation (71). Reduced obesity in adipocyte-P2Y_6_R KO resulted in lower liver weight and hepatic steatosis (71). Further, mRNA levels of inflammatory markers were reduced in the liver of adipocyte P2Y_6_R KO mice (71). Another study revealed that mice lacking P2Y_14_R selectively in adipocytes were protected from obesity and displayed reduced liver weight compared to HFD control mice (72). Liver triglyceride levels were significantly reduced in adipocyte P2Y_14_R KO mice, protecting mice from the development of liver steatosis (72). Reduced obesity and hepatic steatosis further contributed to improved insulin sensitivity in the liver of adipocyte-P2Y_14_R KO mice (72). These studies highlight that blocking P2Y_6_R and P2Y_14_R in adipocytes protects against diet-induced obesity (DIO) and hence has the potential to treat NAFLD/NASH (**Figure 2**).

### Liver Fibrosis

**Figure 3:**
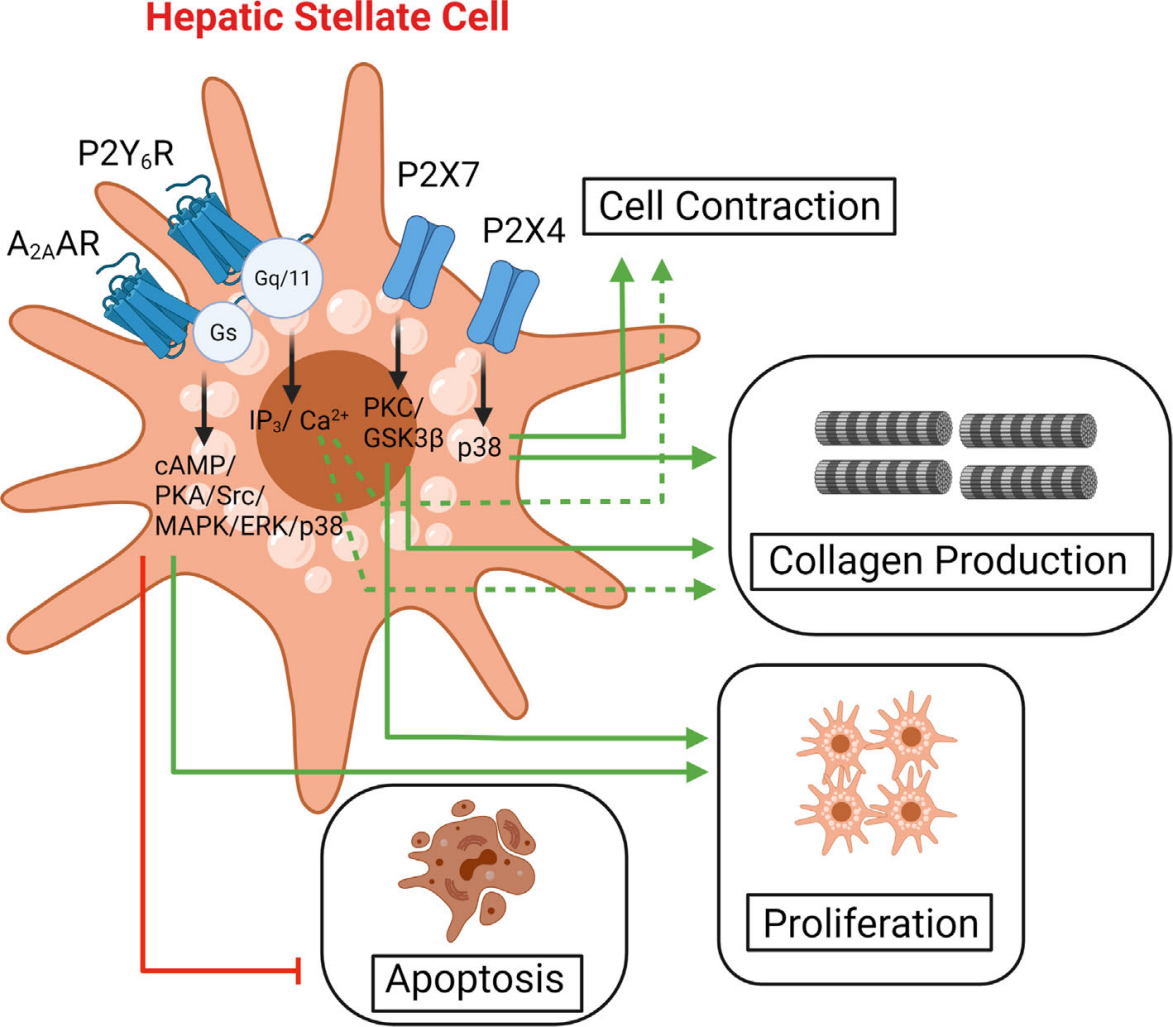
Purinergic signaling in hepatic stellate cells. Hepatic stellate cells (HSC) are classified as non-parenchymal cells of the liver. HSC activation and proliferation results in the secretion of extracellular matrix causing liver fibrosis. Purinergic receptors are expressed in HSC and regulate key processes associated with the initiation and progression of liver fibrosis.

Repetitive injury or inflammation due to NAFLD/NASH causes scarring of liver tissue or fibrosis (73, 74). Untreated fibrosis can lead to irreversible liver damage and progress to liver cirrhosis. Hepatic fibrosis is characterized by the accumulation of extracellular matrix due to activation and differentiation of hepatic stellate cells (HSC) into fibrogenic myofibroblasts (74). Purinergic receptors have been implicated in the regulation of HSC activation. Activation of A_2A_AR induced proliferation and reduced apoptosis and senescence of rat primary HSC and the human HSC cell line LX-2 (**Figure 3**). Mechanistically, A_2A_AR activation down-regulates p53 and retinoblastoma (Rb) protein levels (both tumor suppressors), enhancing HSC survival and contributing to liver fibrosis (75). An A_2A_R antagonist may prove useful in the treatment of ethanol-induced liver fibrosis and HSC activation (76). An A_2B_AR antagonist (MRS1754) has shown promising results in mitigating collagen deposition during hepatic fibrosis progression (77).

Quiescent HSCs with low levels of proliferation and decreased extracellular matrix deposition express P2Y_2_R and P2Y_4_R, whereas activated HSCs expressed P2Y_6_R (78). Treatment of activated HSCs with UDP (native P2Y_6_R agonist) tripled the mRNA levels of procollagen-1, indicating that P2Y_6_R may play a role in liver fibrosis (78) (**Figure 3**). P2X7R expression levels also increased significantly in activated HSCs, promoting proliferation and collagen production (79). P2X7R expression is enhanced in mouse models of liver fibrosis treated with CCl_4_ and treatment with a P2X7R antagonist (A438079) decreased liver inflammation and collagen accumulation (80). P2X4R expression was increased in the MCDD liver fibrosis mouse model (81). Deficiency of P2X4 or treatment with the 5-BDBD (a P2X4R antagonist) protected mice from MCDD-induced liver fibrosis (81).

Activity of CD73, ecto-5′-nucleotidase, was higher in quiescent HSCs than the activated HSCs, indicating that adenosine may play a key role in maintaining the quiescent phenotype of HSCs (82). However, a recent study showed that CD73 expression increased in differentiated myofibroblast and may be targeted for fibrosis treatment (83). Lack of CD73 protected mice against CCl_4_- and thioacetamide (TAA)-induced liver fibrosis (84).

### Liver Cancer

Liver fibrosis and cirrhosis can progress to the development of liver cancer associated with chronic inflammation, dysfunctional metabolism and immune responses, and aberrant cell proliferation (85). ATP released from the necrotic cells acts as a danger signal to activate immune cells during cancer development and stimulate neighboring cells to die. Change in the concentration of extracellular ATP from 1 mM to 2.5 mM tipped the balance from mechanistic target of rapamycin (mTOR)-mediated autophagy required for cell survival to AMP-activated kinase (AMPK)-mediated apoptosis-induced cell death in hepatoma cells (86). This study provides evidence for the manipulation of extracellular ATP for cancer therapy. Extracellular ATP causes increased expression of purinergic receptors in hepatic tumor tissue compared to healthy liver tissue (87–89). P2Y_11_R receptor is expressed at very high levels in human hepatocellular carcinoma (HCC) tissues and was scarcely detected in normal liver tissues (89). P2Y_11_R mediates ATP-induced Ca^2+^ signaling and cell migration in human HCC cells (89). Accordingly, treatment with a P2Y_11_R antagonist (NF340) attenuated the effects of ATP on HCC cells (89). ATP-induced activation of P2Y_2_R mediates the proliferation and migration of human HCC cells (90). Knockdown of P2Y_2_R expression by shRNA inhibited the action of ATP on the cellular behavior of HCC cells (90). These studies indicate that blocking P2Y_11_R and P2Y_2_R signaling may prevent the proliferation and migration of cancerous cells and may be useful for the treatment of liver cancer.

CD39 (ectonucleoside triphosphate diphosphohydrolase-1, ENTPD1) deficiency increases ATP levels activating AMPK and mTOR pathways to stimulate hepatocyte proliferation (91). CD73 is a prognostic marker of HCC as it is expressed highly in around 50% of HCC samples compared to the healthy tissues (92). CD73 activity increase HCC growth and metastasis *via* promoting PI3K/AKT signaling *in vivo* (92, 93). Blocking CD73 with α,β-methylene-ADP (AMPCP) or A_2A_AR with istradefylline (KW6002, now FDA-approved for Parkinson’s disease treatment) inhibited tumor growth (92). Co-treatment with CD73 and A_2A_AR inhibitors displayed synergistic effects on HCC cells (92). High expression of A_3_AR was also reported in tumor tissues and peripheral blood mononuclear cells from patients suffering from HCC (94, 95). Treatment with A_3_AR agonist (CF102) promotes apoptosis and inhibits the growth of HCC cells in a dose-dependent manner (94, 95).

## Conclusions

This review has highlighted the developing role of purinergic receptors in the regulation of hepatic disorders associated with metabolic syndrome. Among adenosine receptors, preclinical studies have highlighted the key role of A_3_AR agonists in protecting against NASH. A_3_AR agonist may also be useful in preventing the growth of liver cancer. A_2A_AR and A_2B_AR antagonists may provide therapeutic benefits against liver fibrosis. Among P2Y receptors, P2Y_6_R or P2Y_14_R antagonist may prove beneficial in preventing NAFLD and hepatic inflammation associated with obesity. P2X7R receptors antagonist can be examined for the treatment of NASH and liver fibrosis. Most of the previous studies have focused on whole-body KO mouse models or pharmacological manipulation for studying purinergic signaling effects on liver metabolism. Studies on understanding the role of purinergic receptors in the liver pathophysiology using liver cell-specific KO mouse are lacking. Future studies using liver-specific KO mouse models for understanding liver diseases are warranted. Numerous potent ligands for purinergic receptors have been synthesized that can be tested in preclinical mouse models of liver diseases (96–103). Characterization of agonists and antagonists for purinergic receptors in preclinical mouse models may foster the development of novel drugs for the treatment of liver diseases.

## Author Contributions

Conceptualization: SJ and KJ. SJ wrote the first draft. Writing: KJ. All authors contributed to the article and approved the submitted version.

## Funding

Support from the NIDDK Intramural Research Program (ZIADK31116, ZIAKD31117) is acknowledged.

## Conflict of Interest

The authors declare that the research was conducted in the absence of any commercial or financial relationships that could be constructed as a potential conflict of interest.

## Publisher’s Note

All claims expressed in this article are solely those of the authors and do not necessarily represent those of their affiliated organizations, or those of the publisher, the editors and the reviewers. Any product that may be evaluated in this article, or claim that may be made by its manufacturer, is not guaranteed or endorsed by the publisher.
